# Mechanistic Understanding of Idiosyncratic Drug-Induced Hepatotoxicity Using Co-Cultures of Hepatocytes and Macrophages

**DOI:** 10.3390/antiox12071315

**Published:** 2023-06-21

**Authors:** Estela Villanueva-Badenas, M. Teresa Donato, Laia Tolosa

**Affiliations:** 1 Unidad de Hepatología Experimental, Instituto de Investigación Sanitaria La Fe, 46026 Valencia, Spain; 2 Departamento de Bioquímica y Biología Molecular, Facultad de Medicina y Odontología, Universidad de Valencia, 46010 Valencia, Spain; 3 Centro de Investigación Biomédica en Red de Enfermedades Hepáticas y Digestivas (CIBERehd), Instituto de Salud Carlos III, 28029 Madrid, Spain; 4 Biomedical Research Networking Center on Bioengineering, Biomaterials and Nanomedicine (CIBER-BBN), Instituto de Salud Carlos III, 28029 Madrid, Spain

**Keywords:** hepatocyte, Kupffer cell, macrophages, idiosyncratic hepatotoxicity, drug, oxidative stress

## Abstract

Hepatotoxicity or drug-induced liver injury (DILI) is a major safety issue in drug development as a primary reason for drug failure in clinical trials and the main cause for post-marketing regulatory measures like drug withdrawal. Idiosyncratic DILI (iDILI) is a patient-specific, multifactorial, and multicellular process that cannot be recapitulated in current in vitro models; thus, our major goal is to develop and fully characterize a co-culture system and to evaluate its suitability for predicting iDILI. For this purpose, we used human hepatoma HepG2 cells and macrophages differentiated from a monocyte cell line (THP-1) and established the appropriate co-culture conditions for mimicking an inflammatory environment. Then, mono-cultures and co-cultures were treated with model iDILI compounds (trovafloxacin, troglitazone) and their parent non-iDILI compounds (levofloxacin, rosiglitazone), and the effects on viability and the mechanisms implicated (i.e., oxidative stress induction) were analyzed. Our results show that co-culture systems including hepatocytes (HepG2) and other cell types (THP-1-derived macrophages) help to enhance the mechanistic understanding of iDILI, providing better hepatotoxicity predictions.

## 1. Introduction

Drug-induced liver injury (DILI) is one of the major causes of acute liver failure and drug attrition in preclinical and clinical phases of drug development, or even of withdrawals or black box warnings when they have already been commercialized [1]. DILI is a multifactorial disease and implies a crosstalk between different liver cell types. Typically, hepatotoxicity is classified into the following two types: intrinsic and idiosyncratic. Intrinsic DILI is dose-dependent and predictable, whereas idiosyncratic DILI (iDILI) occurs only in a low percentage of people exposed to a drug and usually cannot be predicted in preclinical studies since it is dose-independent and relies on different patient-specific factors such as age, sex, genetic factors, obesity, or underlying chronic liver disease [1,2]. Although the incidence of iDILI in the general population is low (19 cases per 100,000) [3], it is a major health problem since it can cause acute liver failure, transplantation, or even death [2].

The liver is mainly composed of hepatocytes, although it also contains non-parenchymal cells such as sinusoidal endothelial cells, Kupffer cells (KCs), or hepatic stellate cells that play important roles and impact the phenotype of hepatocytes [4]. KCs are the largest population of resident macrophages in the body and play a key role in the innate immune response [5]. It was described that the liberation of damage-associated molecular patterns (DAMPs) after the hepatocyte’s death provokes the activation of KCs that secrete pro-inflammatory cytokines, reactive oxygen species (ROS), and other mediators that can accelerate or exacerbate liver damage induced by drugs [6].

Cell-based models provide a relatively inexpensive and fast system that can offer valuable toxicological information in the early stages of drug development. Traditional in vitro models are normally based on monocultures of hepatocytes or hepatoma cell lines; however, recently, more complex multicellular and/or three-dimensional (3D) models were described for hepatotoxicity assessments. Despite the fact that current preclinical strategies predict the hepatotoxicity of many drug candidates, one of the most important gaps in the present test models is iDILI detection, since it can be attributed to the individual characteristics [7].

The pathogenesis of iDILI is complex and multifactorial, and the role of the immune system was extensively described in [8]. For this reason, in order to decipher the mechanisms implicated in iDILI, recently, different models that incorporate inflammatory stimuli or the immune component were proposed for the in vitro assessment of iDILI [9,10,11,12,13]. Most of these models are just based on the use of cytokines, although others use cultures mixed with different cell types. In this sense, basically, the following two different co-culture systems can be defined: (a) indirect culture, where two distinct cell types are in different locations and separated by a semipermeable membrane, and (b) direct co-culture, where both cell types are in the same culture [14]. The direct method allows a better recapitulation of the in vivo microenvironment and detection of the effects of soluble and non-soluble factors [14].

In order to assess the role of immune cells in iDILI, we determined the conditions for the direct co-culture of HepG2 cells and activated macrophages derived from the monocytic cell line THP-1, evaluating the impact of treatment with lipopolysaccharide (LPS) and different cytokines, as well as the timing of these stimuli, cell ratios, and culture conditions on the performance of co-cultures. These two immortalized cell lines that are widely used for in vitro assessments were selected for our co-culture system because of their simple use, availability, and easy translation to high-throughput assays. The gene expression analysis of the key regulators of oxidative stress induction was also comparatively analyzed to elucidate the potential sensitivity of the system for studying drug-induced oxidative stress. To demonstrate the usefulness of the selected test system for studying iDILI, we evaluated the toxicity induced by trovafloxacin (TVX) and troglitazone (TGZ), two drugs that are associated with idiosyncratic hepatotoxicity [15,16], compared to their corresponding non-hepatotoxic compounds levofloxacin (LVX) and rosiglitazone (RGZ), respectively; the effects on viability and differential gene expression were analyzed to understand the role of immune cells and a pro-inflammatory microenvironment and the mechanisms induced by these drugs.

## 2. Materials and Methods

### 2.1. Material

Culture media and complements were purchased from GIBCO (Gibco BRL, Paisley, UK). Fetal bovine serum (FBS) was purchased from Capricorn Scientific GmbH (Ebsdorfergrund, Germany). The test compounds were acquired from Merck (Madrid, Spain). Cytokines were purchased from Peprotech (London, UK). Other chemicals were purchased from Merck.

### 2.2. Cell Culture

#### 2.2.1. Maintenance, Monoculture, and Differentiation of THP-1

THP-1 monocytes (ECACC no. 88081201) were cultured in RPMI supplemented with 10% FBS, 2 mM L-glutamine, 50 U of penicillin/mL, and 50 μg of streptomycin/mL.

**Figure 1:**
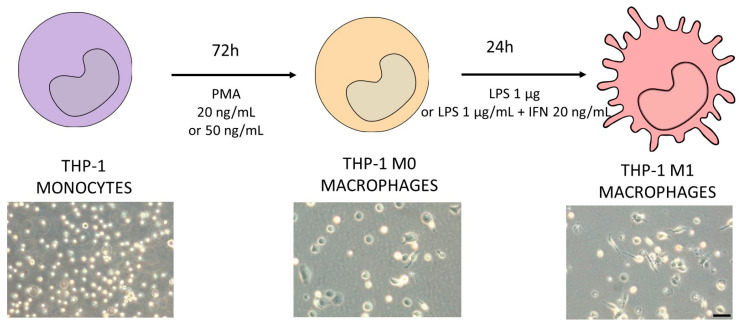
Schematic protocol for differentiating THP-1 monocytes (violet) into M1 macrophages (pink). Different concentrations of PMA were tested in order to decide the optimum protocol to obtain M0 macrophages (orange). The differentiation protocol from M0 to M1 macrophages considered the use of LPS in the presence or absence of IFN-γ. In the bottom of the figure, representative phase-contrast images of each differentiation stage are shown. Scale bar (100 µm) applies to all photographs.

THP-1 cells were differentiated to macrophages via incubation with phorbol-12-myrsitate 13-acetate (PMA, 20 or 50 ng/mL) for 72 h. After differentiation, THP-1 cells became adherent with typical macrophage morphology. To generate M1 macrophages, cells were then stimulated with LPS (1 µg/mL) in the presence or absence of interferon γ (IFN-γ) (20 ng/mL) as described in Figure 1.

#### 2.2.2. Maintenance and Monocultures of HepG2 Cells

HepG2 cells (ECACC No. 85011430) were cultured in Ham’s F-12/Leibovitz L-15 (1:1 *v/v*), supplemented with 7% FBS, 50 U of penicillin/mL, and 50 μg of streptomycin/mL.

HepG2 cells were seeded at a density of 47,000 cells/cm^2^ as a monoculture control for all the experiments.

#### 2.2.3. Co-Culture of HepG2 and THP-1 Macrophages

**Figure 2:**
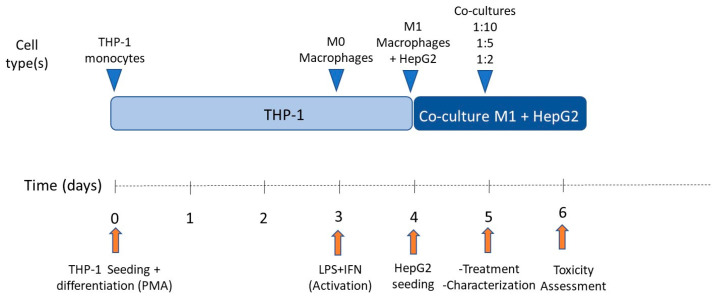
Schedule of co-culture of THP-1 M1 macrophages and HepG2 cells.

For subculturing purposes, cells (HepG2 or THP-1) were detached via treatment with 0.25% trypsin/0.02% EDTA at 37 °C. In order to determine the effect of pro-inflammatory macrophages on HepG2 cells, different co-culture conditions were tested. Co-culture proportions were 1:10, 1:5, and 1:2 (M1:HepG2), respectively. Cells were seeded at 47,000 cells/cm^2^ per condition and co-culture media consisted of HepG2 and M1-THP-1 cell culture media (1:1). The schedule followed for co-culturing HepG2 cells and M1-THP-1 macrophages is exemplified in Figure 2.

For viability studies, cells were seeded in 96-well plates, whereas for gene expression analysis, cells were seeded in 12-well plates.

### 2.3. Characterization of Cell Models

#### 2.3.1. Immunofluorescence

Cells were fixed with 4% formaldehyde for 20 min and washed 3 times with PBS. Then, samples were permeabilized using 0.1% Triton X100 in PBS for 20 min at room temperature, washed with PBS, and blocked with PBS containing 3% BSA for 1 h at room temperature. Then, cells were incubated overnight at 4 °C with the corresponding primary antibodies (Supplementary Table S1) and diluted (1:100) in 1% BSA-PBS. Samples were washed three times and incubated for 1 h with a secondary antibody (1:200, Invitrogen, Madrid, Spain) in 1% BSA-PBS. Then, samples were washed again three times, and finally incubated with Hoechst 33342 (2 µg/mL) for the identification of nuclei. Samples were imaged under a fluorescence microscope (DMI8, Leica Microsystems, Barcelona, Spain). Specificity controls were performed by omitting the primary antibody and resulted in the abolition of immunostaining.

#### 2.3.2. Transcriptomic Analysis

Total RNA was extracted using the RNeasy Plus Mini Kit (Qiagen, Madrid, Spain), and was reverse transcribed as described elsewhere [17]. Diluted cDNA was amplified using LightCycler DNA Master SYBR Green I (Roche Applied Science, Barcelona, Spain) and the appropriate primers (Supplementary Table S2) were amplified using a LightCycler 480 Instrument (Roche Applied Science, Barcelona, Spain). For normalization, porphobilinogen deaminase (PBGD), TATA box binding protein (TBP), Large Ribosomal Subunit Protein UL10 (RPLP0), and actin were used. During each amplification, a reference calibrator cDNA, made from a pool of human livers, was included. The relative mRNA expression was calculated using the LightCycler Relative Quantification Analysis software verson 1.5.0.39. Based on PCR efficiencies and the cycle thresholds, the relative concentration of the target and reference cDNAs and their ratio were determined.

#### 2.3.3. Functionality of Co-Cultures

Urea synthesis was measured using the Quantichrom urea assay kit (BioAssays, Hayward, CA, USA) following the manufacturer’s instructions. The human albumin sandwich enzyme-linked immunosorbent assay (ELISA) quantitation kit (Bethyl Laboratories, Montgomery, TX, USA) was used for measuring albumin production and secretion into the culture media as described by the manufacturer.

### 2.4. Toxicity Studies

For the toxicity studies, HepG2 cells alone or in co-culture with different proportions of M1 macrophages were seeded in 96-well plates (15,000 cells/well) and were allowed to grow and equilibrate for 24 h. Then, cells were exposed for 24 h to eight concentrations of the tested compounds (TVX, LVX, TGZ, RGZ). Each experimental condition was assayed in triplicate wells. Test compounds were diluted in DMSO, and then easily diluted in the culture medium to obtain the desired final concentrations (Table 1). The final DMSO concentration in the culture medium never exceeded 0.5% (*v/v*), and the control cultures were treated with the same amount of solvent.

Cytotoxicity was assessed by means of the tetrazolium reduction colorimetric assay (MTT test) as previously described in detail [19].

Additionally, gene expression analysis of test systems alone or treated with the selected toxic compounds was also performed as indicated in Section 2.3.2. For toxicotranscriptomics studies, cells were seeded in P12-well plates at a density of 47,000 cells/cm^2^.

### 2.5. Statistical Analysis

Data represent triplicate measurements and are expressed as the mean ± SD. Student’s t-test was used for statistical evaluations between two groups, whereas one-way ANOVA followed by Tukey’s multiple comparisons test was used when comparing 3 or more groups. The level of significance was chosen as *p* < 0.05 and calculated using GraphPad Prism v. 8.4.3.

For the multivariate analysis, all the statistical analyses and data plots were run using the free software MetaboAnalyst 5.0 [20]. Principal component analysis (PCA) was used to visualize the natural interrelationship among the samples by performing pairwise comparisons. PLS–DA (projection of latent structures–discriminant analysis) was employed to develop classificatory/predictive models based on the altered patterns aimed to discriminate between cell models alone or treated with TVX or LVX, or with TGZ or RGZ, respectively.

## 3. Results

### 3.1. Differentiation of THP-1 Monocytes into Macrophages

Different protocols for differentiating THP-1 monocytes into macrophages were described in [21]. Generally, there is an induction step to obtain M0 macrophages, followed by a stimulus to obtain M1 or M2 macrophages. In our case, we wanted pro-inflammatory or M1 macrophages for co-culturing purposes; thus, we followed the previously described protocols for differentiating THP-1 cells into M1 polarized macrophages.

**Figure 3:**
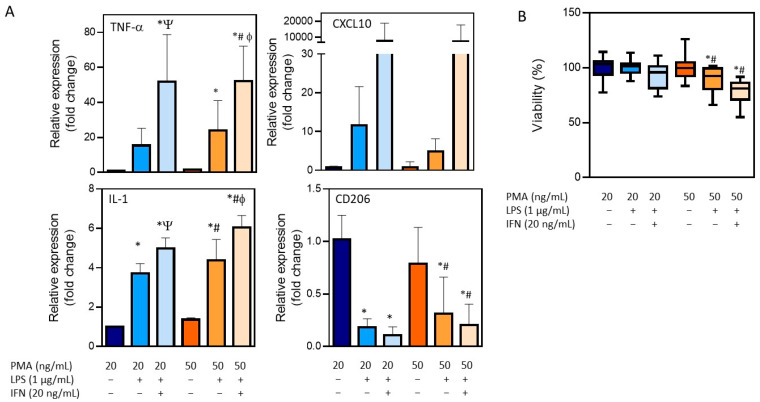
Characterization of the differentiation of THP-1 monocytes into pro-inflammatory macrophages. (**A**) The mRNA expression of M1 (TNF-α, IL-1, and CXCL10) and M0/M2 (CD206) markers after distinct differentiation protocols. (**B**) Viability after the differentiation process following distinct conditions. * At least *p* ≤ 0.01 (compared to PMA 20 ng/mL); ^#^ *p* ≤ 0.01 (compared to PMA 50 ng/mL); ^Ψ^ *p* ≤ 0.01 (compared to PMA 20 + LPS); ^ϕ^ *p* ≤ 0.01 (compared to PMA 50 + LPS) (ANOVA followed by Tukey’s multiple comparisons test).

The treatment of THP-1 cells with different concentrations of PMA resulted in the adherence of cells to the cell culture plastic and the acquisition of a stellate morphology (Figure 1). After priming with PMA, cells were then stimulated with LPS (1 µg/mL) alone or in combination with IFN-γ (20 ng/mL), and the expression of different macrophage markers was comparatively analyzed (Figure 3). The treatment with LPS and IFN-γ resulted in an increased expression of TNF-α, IL-1, and CXCL10, independently from the initial priming stimulus, although it was only statistically significant for TNF-α and IL-1. The use of LPS alone resulted in lower expression levels of the M1 macrophage markers compared to the synergic effect of IFN-γ with LPS. The expression of the M0/M2 marker CD206 was also analyzed. As expected, a significant decrease with respect to the M0 macrophages (THP-1 cells treated with PMA) was observed in different conditions. Since no very significant differences between the different stimulations was found, the effects on cell viability were also studied to define the optimum protocol (Figure 3B). The treatment of THP-1 cells with the highest concentration of PMA (50 ng/mL) for differentiation followed by the treatment with LPS and IFN-γ for obtaining pro-inflammatory macrophages resulted in a significant reduction in the viability (79 ± 5) that led us to select the protocol with the lowest concentration of PMA (20 ng/mL) for priming, followed by a synergic activation with LPS and IFN-γ.

### 3.2. Optimization of Cell Co-Culture Conditions

The next step was to co-culture the HepG2 cells and activate the M1-THP-1 cells. We previously demonstrated that the THP-1 cells maintain an activated state after stimulation with LPS and IFN-γ, and before co-culturing with HepG2 cells, we decided to study whether the pro-inflammatory stimuli should be maintained, or if it could be removed for the co-culture. For this reason, we studied the expression of the activation of M1 markers after stimulation and the effects of removing LPS and IFN-γ after 24 and 72 h. The removal of LPS and IFN-γ resulted in a significant reduction in the expression of the pro-inflammatory markers (TNF-α, IL-1, and CXCL10), whereas the maintenance of the stimuli for 72 h resulted in significant enhanced levels of the same markers. Since it seemed to be necessary to maintain the pro-inflammatory stimuli, we then studied the effects of LPS and IFN-γ in the HepG2 cells. No significant effects on the viability were observed (Supplementary Figure S1); thus, this pro-inflammatory stimuli was kept for the co-cultures of the THP1-M1 and HepG2 cells.

**Figure 4:**
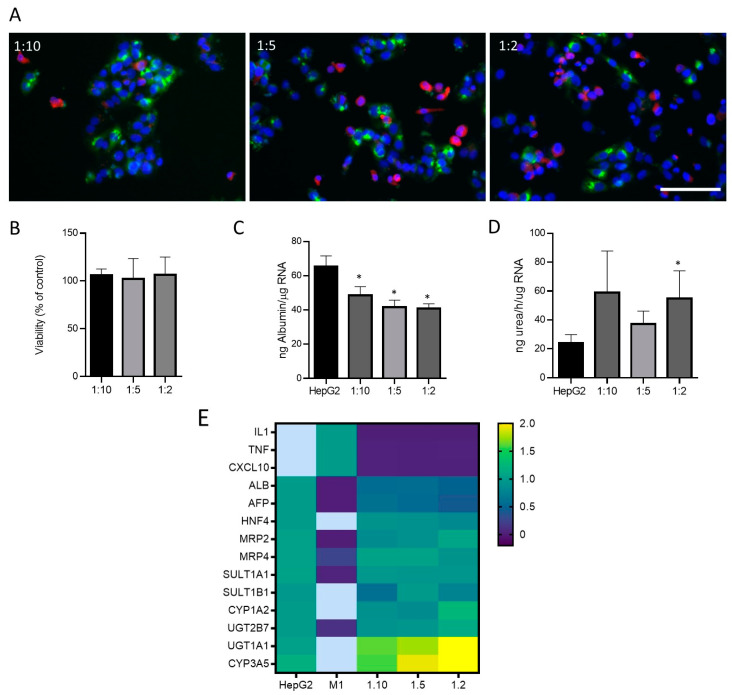
Co-cultures of HepG2 cells and M1-THP-1 cells. (**A**) Immunofluorescence microscope image for albumin (green) and CD68 (red) in co-cultures of HepG2 cells and M1-THP1 cells at different proportions. Nuclei were stained with Hoechst 33342 (blue). Scale bar (100 µm) applies to all photographs. Cell viability (**B**), albumin production (**C**), ureogenic capability (**D**), and mRNA expression (**E**) were determined in co-cultures at different ratios. Heatmap visualization of RT-qPCR analysis of mRNA levels in different test systems are represented as the mean of fold change. Relative quantification of each gene expression level was normalized according to the housekeeping genes. Non-detected samples are represented in light blue. * At least *p* ≤ 0.01 (compared with HepG2 monocultures; ANOVA followed by Tukey’s multiple comparisons test).

Then, different proportions of cells (M1-THP-1:HepG2:; 1:2, 1:5, 1:10) were seeded to simulate a physiological and inflammatory microenvironment, respectively. Immunofluorescence allowed for the identification of cells expressing hepatocyte (HepG2) and M1 markers in different co-culture conditions (Figure 4A). As the proportion of M1 cells increased in the co-culture, more CD68-positive cells could be identified (Figure 4A). The viability was assessed in different conditions with no significant effects in any of the proportions studied (Figure 4B). Additionally, some specific hepatic functions such as albumin synthesis and secretion and ureogenic capability were analyzed and compared to the monocultures of the HepG2 cells (Figure 4C,D). The co-cultures showed a decreased production of albumin as expected, since albumin synthesis is a specific hepatocyte function, and co-cultures have a lower proportion of HepG2 cells. Regarding the ureogenic capability, only the co-cultures with the smallest quantity of M1 macrophages showed a significant increase (Figure 4D). The gene expression analysis of the hepatic markers and M1 markers revealed that the co-cultures showed an increased expression of the key hepatocyte markers such as UGT1A1 or CYP3A5, whereas the M1 markers were only expressed in the M1 cells and showed decreased levels in the co-cultures at different ratios. Other hepatic markers maintained their expression 24 h after the co-culture (Figure 4E).

### 3.3. Modulation of Oxidative Stress Enzymes Expression in Co-Cultures of HepG2 and M1-THP-1 Cells

**Figure 5:**
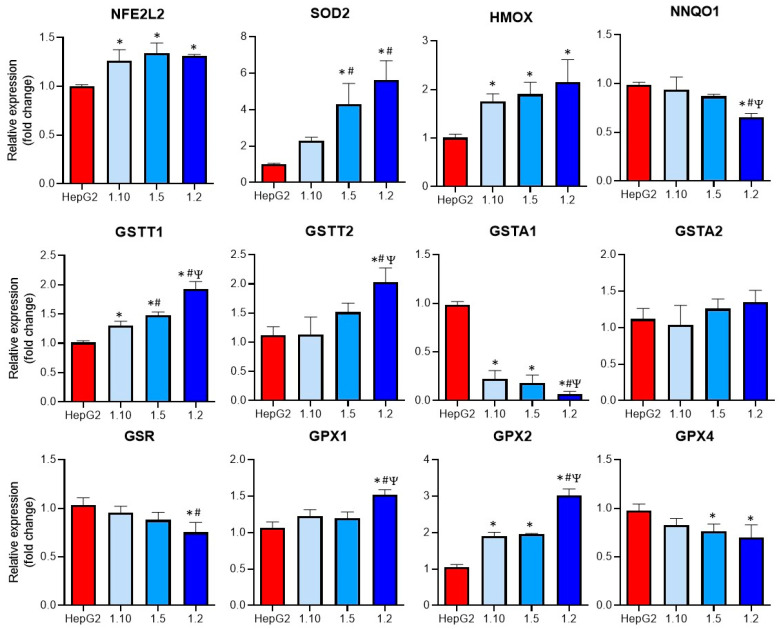
Expression of oxidative-related enzymes in co-cultures of HepG2 cells and M1-THP-1 cells compared to monocultures of HepG2 cells. The mRNA expressions of NFE2L2, SOD2, HMOX, NNQO1, GSTT1, GSTT2, GSTA1, GSTA2, GSR, GPX1, GPX2, and GPX4, were analyzed in monocultures of HepG2 or M1-THP-1 cells and co-cultures of both cell types at different cell ratios. * At least *p* ≤ 0.01 (compared to HepG2); ^#^ *p* ≤ 0.01 (compared to co-cultures HepG2:M1-THP-1 (ratio 1:10)); ^Ψ^ *p* ≤ 0.01 (compared to co-cultures HepG2:M1-THP-1 (ratio 1:15)); (ANOVA followed by Tukey’s multiple comparisons test).

Oxidative stress was described as a major mechanism implicated in DILI and also plays an important role in KC activation [22,23,24]. For this reason, the mRNA expression of different enzymes implicated in the GSH metabolism and in the induction of oxidative stress was comparatively analyzed in the monocultures of the HepG2 cells and the co-cultures (Figure 5).

Increased significant levels of GSTT1, GPX2, NFE2L2, and HMOX mRNAs were observed in all the co-cultures, whereas some of the analyzed genes (GSTT2, SOD2, GPX1) only showed significant differences at the co-cultures with the higher proportion on the M1 cells (1:2). The expression of GSTA1 was significantly reduced in all the co-culture conditions, although the highest reduction compared to the other co-cultures was observed at a 1:2 ratio. When the expression of GPX4 was studied, a significant decrease in the 1:5 and 1:2 co-cultures compared to the monocultures of HepG2 was observed. Finally, the GSR expression was significantly reduced in the co-cultures with the higher proportion of activated macrophages. These differences could increase the sensitivity of co-cultures to hepatotoxicants that induce oxidative stress.

### 3.4. Co-Cultures Exhibit Different Sensitivity to Hepatotoxicants (Pro-Inflammatory Macrophages Increase Trovafloxacin-Induced Toxicity)

In order to study the suitability of the developed test systems for the iDILI evaluations, the cells were incubated with different concentrations of TVX or TGZ, two iDILI drugs, or their non-DILI analogues LVX or RGZ. Previously, we verified the absence of cytotoxic effects in HepG2 cells due to LPS and IFN-γ (Supplementary Figure S1); thus, we performed the hepatotoxicity studies in the presence of these stimuli to maintain the activation of the macrophages. HepG2 cells in the monoculture were always used in parallel to determine whether the pro-inflammatory factor alone was able to produce synergistic effects.

**Figure 6:**
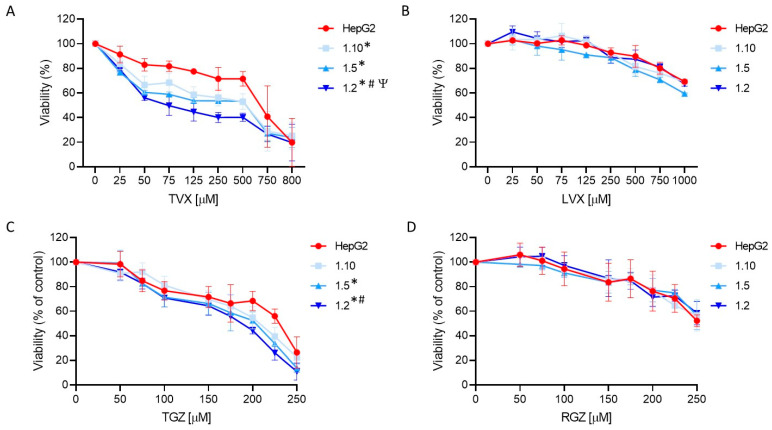
Concentration-dependent toxicity of test compounds in HepG2 monocultures and different co-cultures of HepG2 and M1-THP-1 cells. HepG2 monocultures or co-cultures at different cell ratios were exposed for 24 h to TVX (**A**), LVX (**B**), TGZ (**C**), or RGZ (**D**), and viability was assessed. Data are expressed as the percentage of vehicle (DMSO)-treated cells and represent the mean ± SD of at least 3 independent experiments. * At least *p* ≤ 0.005 (compared to HepG2); ^#^ *p* ≤ 0.01 (compared to 1:10 co-cultures); ^Ψ^ *p* ≤ 0.01 (compared to 1:5 co-cultures) (ANOVA followed by Tukey’s multiple comparisons test).

TVX induced a significant cell death at concentrations higher than 50 µM (Figure 6). A synergistic effect was detected in the co-cultures exposed to TVX compared to the monocultures of the HepG2 cells. Additionally, an increased sensitivity was observed in the co-cultures with a major proportion of macrophages, indicating that an inflammatory component influences TVX-induced hepatotoxicity. ANOVA followed by Tukey’s test revealed significant differences among the different cell systems treated with TVX. The IC50 values for all the test systems used also revealed differences, since the 1:2 co-cultures showed the lowest IC50 value (74 µM) (Supplementary Table S3). By contrast, LVX only exhibited a significant effect at the greatest concentrations (>750 µM), and no synergic effect was observed in the co-cultures with macrophages. These results demonstrate the advantages of employing co-cultures of hepatocytes and macrophages to increase the sensitivity of in vitro hepatotoxicity assessments.

TGZ also produced significant cell death at concentrations higher than 100 µM, and the decrease in the viability was more significant in the co-cultures at the highest ratios of M1 cells (1:5 and 1:2, ANOVA, post-Tukey) (Figure 6C). The IC50 values also confirmed an increased sensitivity of the 1:2 co-cultures since they exhibited the lowest value (188 µM) compared to the monocultures of the HepG2 cells (223 µM) (Supplementary Table S3). By contrast, RGZ produced a significant decrease in the viability at a concentration higher than 200 µM, and no significant differences between the test systems were found (Figure 6D).

### 3.5. Mechanistic Understanding of Idiosyncratic Drug-Induced Toxicity

After detecting significant differences in the TVX- and TGZ-induced toxicity among the different culture conditions, we investigated whether there were differences in the expression of genes that were related to this hepatotoxicity. The mRNA levels of the Nfr2 pathway, GSH-related enzymes, and apoptotic genes were analyzed.

**Figure 7:**
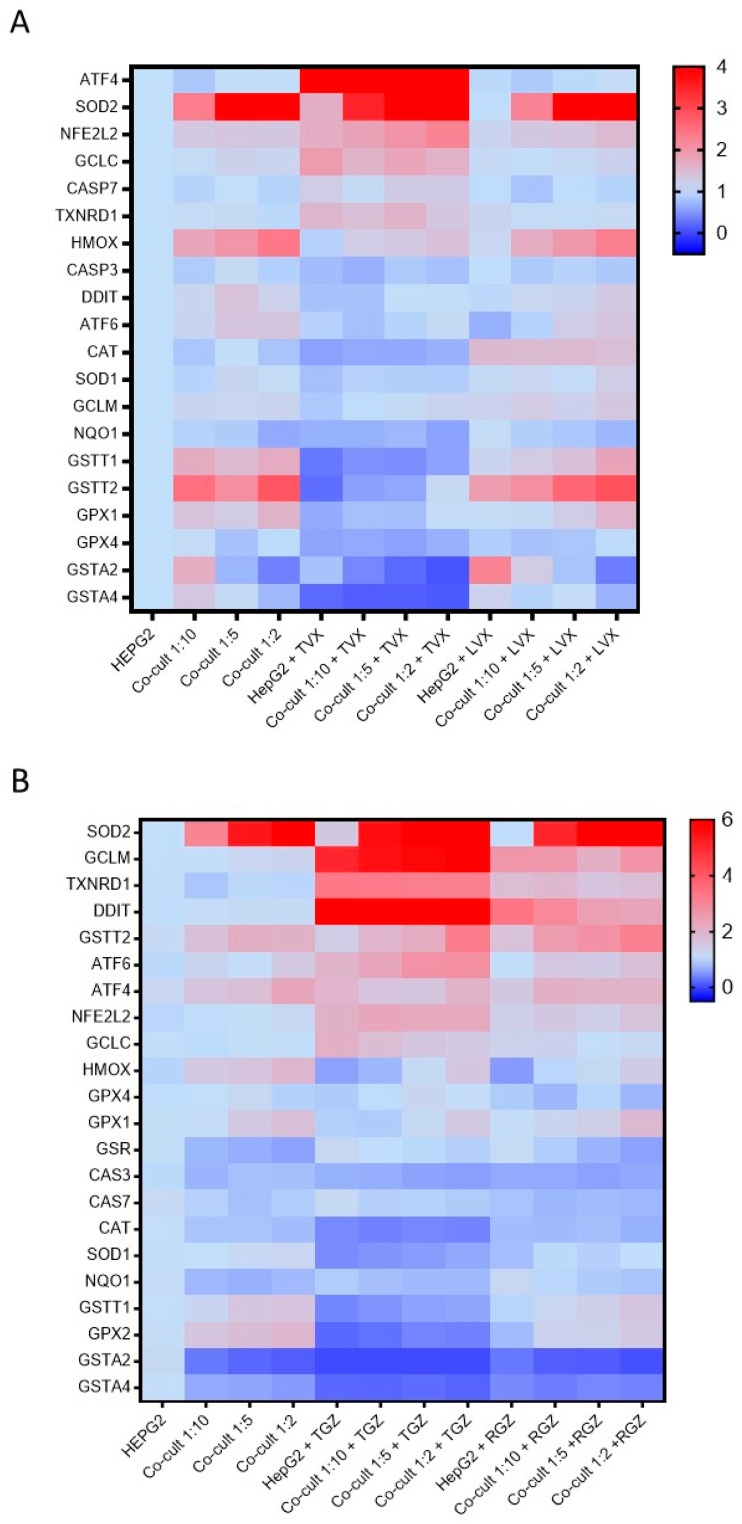
Differences in gene expression profiles in monocultures and co-cultures exposed to hepatotoxic compounds or their analogues. The mRNA levels of 22 genes implicated in iDILI were measured in HepG2 cells and co-cultures at different ratios (1:10, 1:5, and 1:2) exposed to TVX (25 µM) or LVX (25 µM) for 24 h (**A**), or TGZ (100 µM) or RGZ (100 µM) (**B**).

TVX reduced the GSTT1, GSTT2, GSTA2, GSTA4, NNQO1, GPX1, GPX2, and GPX4 levels in all the culture conditions. Increased levels of AT4 and SOD2 were also observed in the TVX-treated cells (Figure 7A). No significant differences among the HepG2 cells or the different co-cultures were observed. There were similar significant increases in the expression of the ATF4, SOD2, NFE2L2, and GCLC levels, independent from the cell model tested (Supplementary Figure S2). In addition, LVX did not significantly change the gene expression compared to the non-treated cultures.

TGZ increased the GCLM, DDIT, and TXNRD1 levels and reduced the GSTA2, GSTA4, GSTT1, and GPX2 levels compared to the non-treated cells, although the response to the toxicant was similar in the monocultures and co-cultures (Figure 7B). When the response to the toxicant was analyzed and normalized by the cell type, significant and similar increases in SOD2, GCLM, TXNRD1, and DDIT were observed, although RGZ also produced increases in the expression of GCLM, TXNRD1, and DDIT (Supplementary Figure S3). For the other genes, RGZ showed a gene expression pattern similar to the non-treated cells.

**Figure 8:**
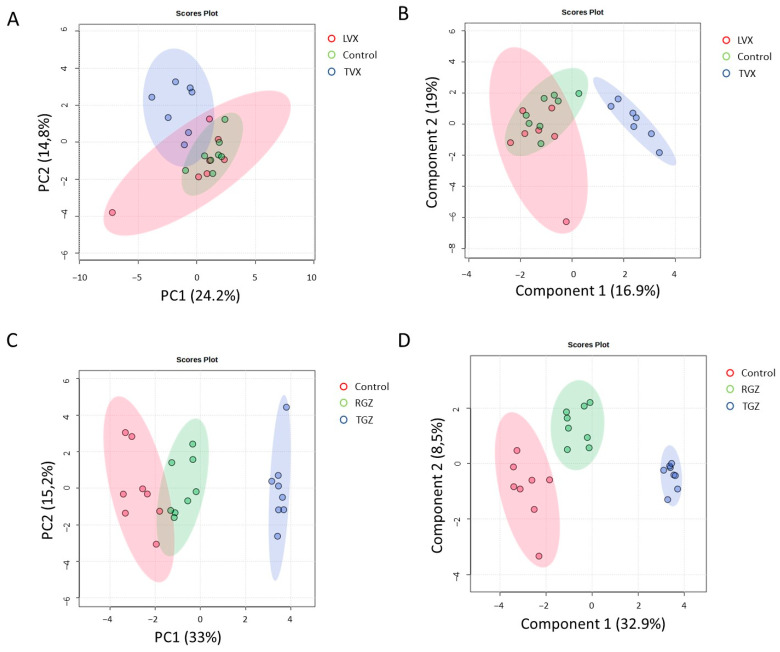
Multivariate analysis of gene expression analysis of treated and untreated cultures. PCA (**A**,**C**) and PLS–DA (**B**,**D**) analysis of gene expression grouped by treatment with TVX and LVX (**A**,**B**) or TGZ and RSG (**C**,**D**) compared to untreated cultures. Blue clusters are test systems treated with test iDILI compounds (TVX or TGZ, respectively).

A multivariate analysis revealed that the cultures treated with TVX clustered together and separated from the non-treated and LVX-treated cells (Figure 8A,B), which indicates a unique pattern of pathways modulated by TVX in all culture systems. Similar results for PCA and PLS–DA were observed in the case of the test systems treated with TGZ, whereas the cultures treated with RGZ were more similar to the untreated cultures (Figure 8C,D).

## 4. Discussion

There is an evident shortage of knowledge in iDILI detection and diagnosis; thus, new models that allow for a better prediction and understanding of the underlying mechanisms are urgently needed to diminish the risks in the drug development process [25]. Although the immune cells were implicated in the iDILI pathogenesis, there is no standard method for identifying compounds that could produce immune-mediated iDILI [25]. Here, we fully characterized a direct co-culture system for the detection of immune-mediated iDILI drugs that could easily be adaptable as a screening tool for high-throughput assays for the early detection of iDILI compounds. Other authors have used other human cell models such as primary KCs and hepatocytes for studying the influence of the immune component in iDILI [13,26]; however, the scarcity of liver tissue for obtaining primary cells, their phenotypic instability, and the short life span of primary human hepatocytes limit their wider application for DILI evaluations.

We explored the role of classically activated macrophages in iDILI by establishing a direct co-culture method using HepG2 cells and differentiated M1-THP-1 cells. THP-1 cells are a pro-monocytic cell line that can differentiate into macrophages when stimulated with PMA [21]. Macrophages are classified as classically or alternatively activated cells (M1 or M2, respectively) that exhibit distinct features [21,27]. In this sense, diverse differentiation protocols were described. We initially characterized the differentiation process and the stimuli necessary to keep the pro-inflammatory phenotype of M1-THP-1 cells before co-culturing them with hepatic cells. Our results demonstrate that it is necessary to maintain the stimuli to have M1 macrophages that exhibit pro-inflammatory characteristics needed for mimicking what happens in vivo.

Then, we explored different proportions of activated macrophages with HepG2 cells and their possible role in iDILI-induced toxicity. Other studies used indirect co-culture (in which different cell types are separated by a permeable membrane) for studying the effects of the factors such as cytokines released by KCs (or THP-1 macrophages) on hepatocytes [12,27] or even the use of conditioned media from promyelocytic cells [28]. However, the direct co-culture of HepG2 cells and activated macrophages would better mimic the crosstalk that occurs in vivo. A total of 24 h after seeding, the co-cultures maintained the expression of both the hepatocyte markers and pro-inflammatory markers, and also maintained some specific hepatic functions such as ureogenesis and albumin synthesis. Although different studies demonstrated that non-parenchymal cells increase hepatic functionality, particularly in long-lasting cultures [4,29], the developed systems did not show improvements in the albumin synthesis or urea production. This could be due to the fact that the functionality study was assessed very early (24 h) after the HepG2 seeding.

The liver is very immunological and includes a huge amount of innate and adaptative immune cells that maintain local homeostasis and control different pathological processes [30]. It is known that particular types of DILI, such as iDILI, in which inflammation was implicated, can be more precisely modeled if the test systems incorporate Kupffer cells in addition to hepatocytes [4]. The direct co-culture system described here exhibited an enhanced toxicity to TVX and TGZ, and this increased susceptibility was directly correlated to the amount of M1 cells, which would indicate a role in the hepatotoxicity induced by these drugs. In this sense, an inflammatory microenvironment was associated with an augmented susceptibility to iDILI [9,31,32], although the precise mechanisms underlying this phenomenon are unknown. Mechanistic toxicology is currently fundamental in the preclinical studies of the pharmaceutical companies since it provides better predictions, a reduction in side effects, as well as the discovery and application of new DILI biomarkers [33]. In this sense, we examined the possible mechanisms implicated in TVX- and TGZ-induced toxicity.

An alteration in the redox homeostasis can cause enhanced reactive oxygen species (ROS) levels that may have detrimental effects on cells such as oxidative damage to protein, lipids, or DNA [34]. Oxidative stress induction due to the inhibition of detoxification enzymes or produced by a disproportionate ROS production was implicated in DILI susceptibility and the level of damage [35]. The co-cultures exhibited a different profile in the expression of GSH-related enzymes that could explain the susceptibility to iDILI drugs. On the other hand, innate immunity can be triggered by DAMPs released by damaged hepatocytes, causing inflammation [36]. In fact, it was described that when some iDILI drugs are incubated with hepatocytes, the released DAMPs are able to activate THP-1 inflammasomes [37]. In our system, the possible role of DAMPs should be also considered as a reason for the increased sensitivity to iDILI drugs.

Antibiotics were described as a common cause of iDILI [38]. TVX is fluoroquinolone antibiotic, released in 1998, that received a black box warning in 1999 after being associated with serious hepatotoxicity cases [15]. When used in vivo, TVX synergizes with LPS, resulting in hepatotoxicity in rats and mice [39,40]. When used in vitro, TVX also interacts with LPS and/or other pro-inflammatory cytokines such as TNF-α [9,11,31,39,41] to produce the apoptotic death of hepatocytes. By contrast, LVX is another fluoroquinolone that was not described to produce iDILI [38] and does not show the synergistic effect with pro-inflammatory stimuli [41]. Although different mechanisms were proposed and analyzed independently in different in vivo [42] and in vitro models [9,12,42], a full mechanistic understanding of human in vitro test systems that consider the crosstalk between different cell types was not described. Recently, Yang and colleagues (2022) used RNAseq technology for analyzing the transcriptomic changes of KCs treated with TVX and identified an upregulation of genes related to inflammatory and immune responses [27]. Our results show that a modulation of different genes implicated in oxidative stress is induced by TVX and not by its analogue LVX in monocultures and co-cultures. Although differences in the gene expression of GSH enzymes was clearly shown, these differences are not responsible for TVX-induced toxicity, since the modulation is similar in the different co-cultures (Figure 7A).

Another explanation for the increased susceptibility of co-cultures to TVX could be the differences in the UGT1A1 expression, since UGT1A1 was implicated in TVX metabolism [43]. In fact, Mitsugi and colleagues showed that the induction of UGT1A1 in HepG2 cells resulted in increased toxicity when treated with TVX compared with cells only treated with TVX [44].

On the other hand, troglitazone is an antidiabetic drug that was withdrawn from the market due to severe iDILI cases [16]. Different case reports described the implication of the immune system in TGZ-induced toxicity in humans [45,46] and, in some cases, treatment with corticosteroids decreased the response in patients [45,47]. Oxidative stress and mitochondrial injury were suggested as major mechanisms implicated in TGZ-induced hepatotoxicity using in vitro and genetically modified mice models [48,49,50].

The gene expression analysis showed significant differences for key oxidative stress markers (i.e., SOD2, DDIT/CHOP, GCLM) after treatment with TGZ, although no significant differences between the monocultures and co-cultures that could explain the increased susceptibility was detected (Figure 7B). Previous studies showed that oxidative stress plays a significant role in TGZ-induced toxicity [51]. Additionally, endoplasmic reticulum stress and the accumulation of the pro-apoptotic protein CHOP was also implicated and was linked to DILI pathogenesis [52]; thus, CHOP up-regulation could be a consequence of endoplasmic reticulum stress induced by TGZ in hepatic cells. In fact, Edling et al. (2009) demonstrated that TGZ induced the expression of different stress-related mRNAs such as DDIT3/CHOP or CAT in an indirect co-culture of THP-1 and Hu7 cells [53].

For a more in-depth mechanistic understanding of iDILI, other concentrations and timepoints could possibly provide more information about the implicated pathways. Other studies based on the identification of specific cell responses such as immunofluorescence-based assays or single-cell studies could help to elucidate the specific cell responses that lead to iDILI.

Finally, although the co-culture system described here is helpful for understanding cell–cell crosstalk and mimics better in vivo physiology, the use of non-physiological conditions (i.e., test compound concentrations) and the decreased metabolic capacity of the model compared to the in vivo conditions should also be considered when deciphering the results [54]. The use of complementary approaches such as in silico methods could offer a broader view for understanding iDILI.

## 5. Conclusions

We have shown the utility of a direct co-culture of hepatocytes and polarized macrophages for exploring the mechanisms implicated in iDILI. This system responds to prototype hepatotoxic compounds and shows enhanced immune-mediated effects. In fact, the developed cell culture model allows us to mimic both a physiological and inflammatory microenvironment, which enables us to evaluate the role of the immune component in iDILI. The dialog between the hepatocytes and immune cells was described to play a major role in iDILI [25]; however, since it is a patient-specific process, personalized models, for instance, derived from induced pluripotent stem cells, may offer an approach that could better reflect how the signals released by hepatocytes affect the immune component.

## Figures and Tables

**Table 1:** Test compounds.

Compound	CAS Number	Supplier	Concentrations (µM)	Cmax (µM) [18]	iDILI Classification
Trovafloxacin	147059-72-1	Merck	25–800	4.1	+
Levofloxacin	100986-85-4	Merck	25–1000	15.7	−
Troglitazone	97322-87-7	Merck	50–250	6.4	+
Rosiglitazone	122320-73-4	Merck	50–250	1	−

## Data Availability

Data are available upon reasonable request.

## Supplementary Materials

The following supporting information can be downloaded at https://www.mdpi.com/article/10.3390/antiox12071315/s1, Supplementary Figure S1: Characterization of M1 macrophages under pro-inflammatory conditions during 72 h; Supplementary Figure S2: Expression of oxidative and apoptotic-related mRNA in cell culture models exposed to TVX or LVX; Supplementary Figure S3: Expression of oxidative and apoptotic-related mRNA in cell culture models exposed to TGZ or RGZ; Table S1: Antibodies used in the study; Table S2: The oligonucleotides used for quantitative RT-PCR; Table S3: IC50 values for test compounds in different in vitro models.

## Abbreviations

3D: three-dimensional; DAMPs: damage-associated molecular patterns; DILI: drug-induced liver injury; FBS: fetal bovine serum; iDILI: idiosyncratic DILI; IFN-γ: interferon gamma; KCs: Kupffer cells; LPS: lipopolysaccharide; LVX: levofloxacin; PCA: principal component analysis; PLS–DA: projection of latent structures–discriminant analysis; PMA: phorbol-12-myrsitate 13-acetate; RGZ: rosiglitazone; ROS: reactive oxygen species; TGZ: troglitazone; TVX: trovafloxacin.
