# Supplementary material for: Mechanistic Understanding of Idiosyncratic Drug-Induced Hepatotoxicity Using Co-Cultures of Hepatocytes and Macrophages
Source: Antioxidants (Basel). 2023 Jun 21;12(7):1315. doi: 10.3390/antiox12071315 (PMC10376129; doi:10.3390/antiox12071315)
Supplement: Supplementary file 1 [file antioxidants-12-01315-s001.zip › antioxidants-2456568-supplementary.pdf]

SUPPLEMENTARY MATERIAL Villanueva-Badenas et al., 2023

SUPPLEMENTARY FIGURES

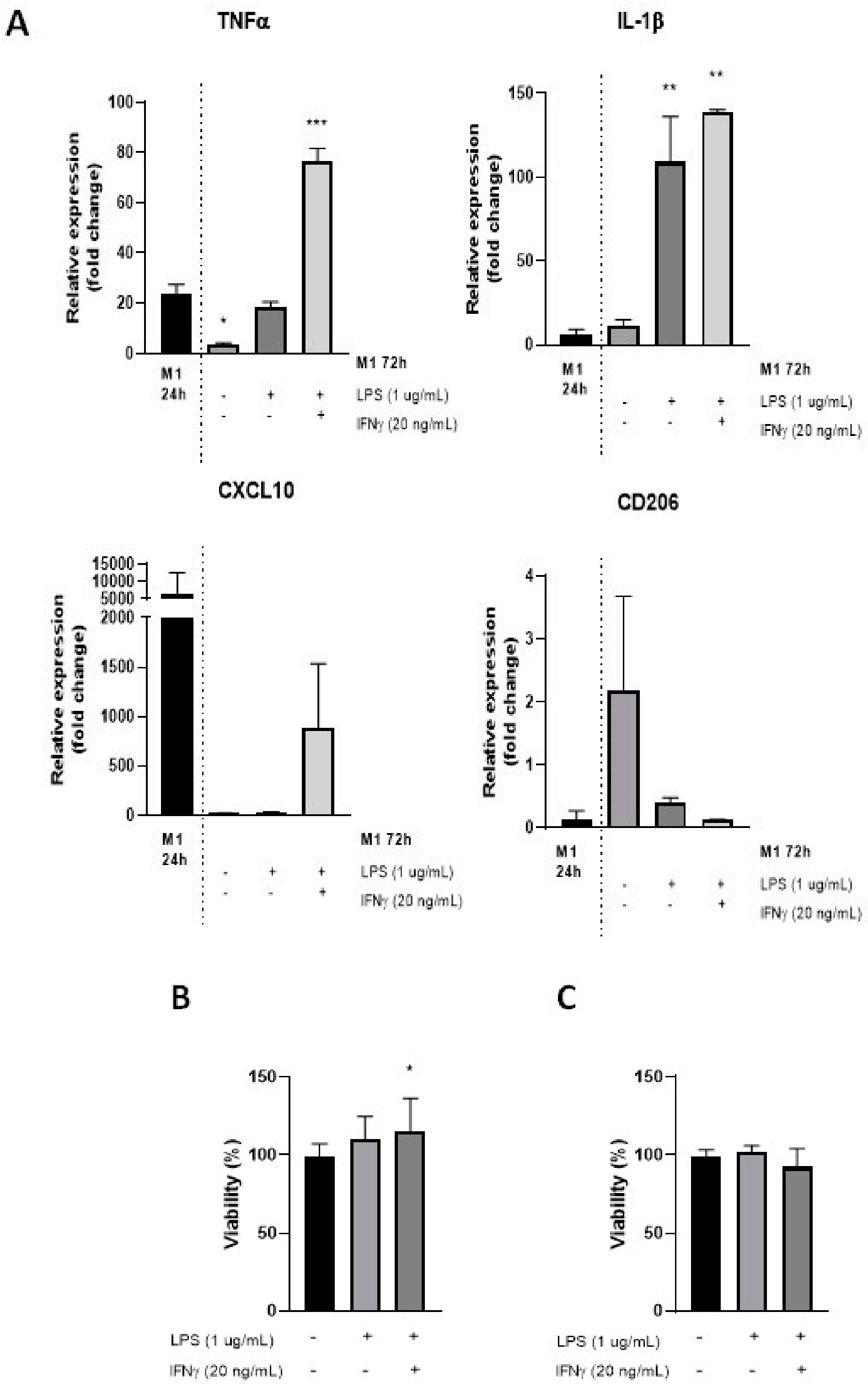

Supplementary Figure S1: **Characterization of M1 macrophages under pro-inflammatory conditions during 72 h**. (A) mRNA expression of M1 (TNF-α, IL-1 and CXCL10) and M2 (CD206) markers after proinflammatory stimulation during 72 h. (B) HepG2 viability after proinflammatory stimulation during 72 h. (C) M1 viability after proinflammatory stimulation during 72 h. *At least $p \leq 0.01$ (compared to M1 24 h or HepG2 24 h)

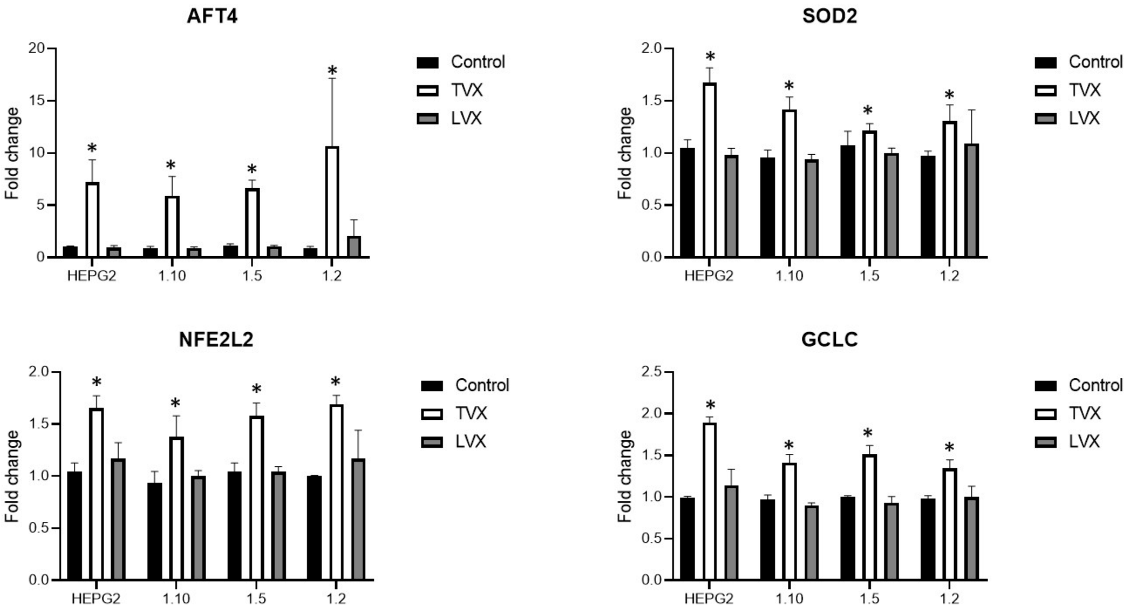

Supplementary Figure S2. Expression of oxidative and apoptotic-related mRNA in cell culture models exposed to TVX or LVX. mRNA expression of AFT4, SOD2, NFE2L2 and GCLC was analysed in monocultures of HepG2 or and co-cultures of HepG2 cells and M1-THP1 cells at different cell ratios exposed to 25 µM of TVX or LVX and compared to non-treated cultures (Control). *At least p≤0.01 (compared to non-treated cultures, Student's t-test).

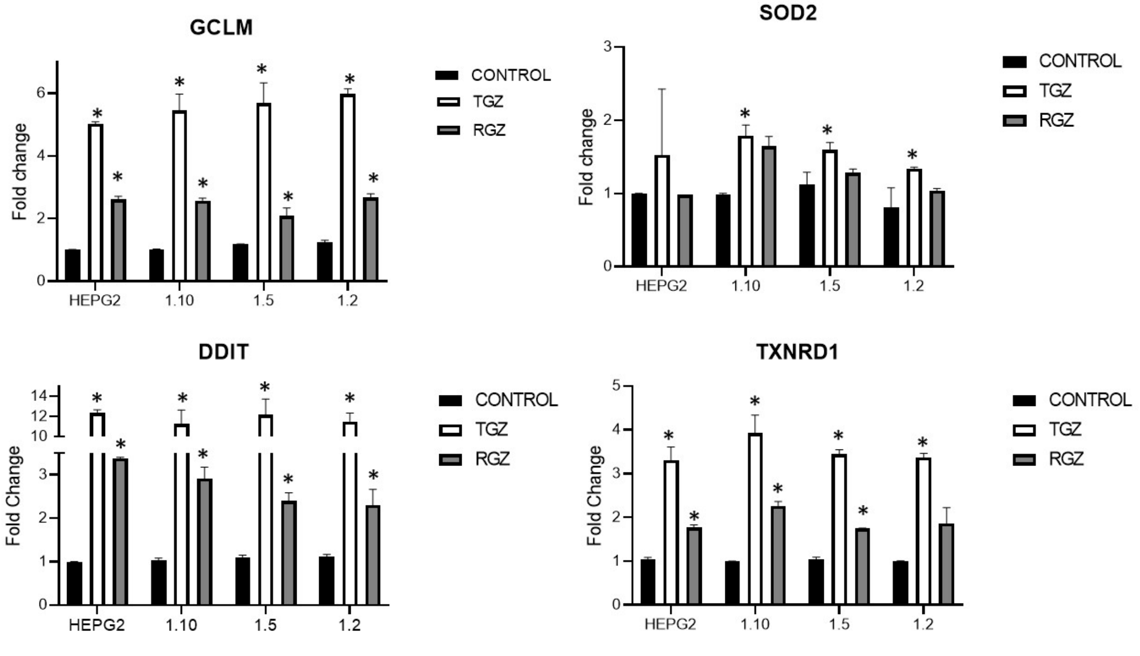

Supplementary Figure S3. Expression of oxidative and apoptotic-related mRNA in cell culture models exposed to TGZ or RGZ. mRNA expression of GCLM, SOD2, DDIT and TXNRD1 was analysed in monocultures of HepG2 or and co-cultures of HepG2 cells and M1-THP1 cells at different cell ratios exposed to 100 µM of TGZ or RGZ and compared to non-treated cultures (Control). *At least p≤0.01 (compared to non-treated cultures, Student's t-test).

SUPPLEMENTARY TABLES

Supplementary Table S1. Antibodies used in the study.

**Target**	**Primary Antibody**	**Secundary Antibody**
Albumin	Goat Anti-Human Albumin<br>A80-229ª Bethyl	Alexa Fluor 488 donkey anti-goat<br>A11055 invitrogen
CD68	Recombinant Anti-CD68 antibody [KP1]<br>ab233172 abcam	Alexa Fluor 594 donkey anti-mouse<br>R37115 invitrogen
CD163	Recombinant Alexa Fluor® 488 Anti-CD163 antibody<br>ab218293 abcam	Alexa Fluor 488 donkey anti-rabbit<br>A21202

Supplementary Table S2: The oligonucleotides used for quantitative RT-PCR

Symbol	Gene	Forward (5'->3')	Reverse (5'->3')	RefSeq	nt position
HMBS	hydroxymethylbilane synthase	CGGAAGAAAACAGCCCAAAGA	TGAAGCCAGGAGGAAGCACAGT	NM_000190.4	185-478
TBP	TATA-box binding protein	AAAATGGTGTGCACAGGAGCC	CACATCACAGCTCCCCACCAT	NM_003194.5	895-1035
ACTB	Actin beta	CGTACCACTGGCATCGTGAT	GTGTTGGCGTACAGGTCTTTG	NM_001101.5	523-974
RPLP0	Ribosomal Protein Lateral Stalk Subunit P0	TGGTCATCCAGCAGGTGTTCGA	ACAGACACTGGCAACATTGCGG	NM_001002.4	637-755
ALB	Albumin	CACAGAATCCTTGGTGAACAGG	TGCGAAATCATCCATAACAGC	NM_000477.7	1544-1769
HNF*4A*	Hepatocyte nuclear factor 4 alpha	GCCTACCTCAAAGCCATCAT	GACCCTCCCAGCAGCATCTC	NM_000457.6	993-1267
AFP	Alpha-fetoprotein	GCTTGGTGGTGGATGAAACA	TCCTCTGTTATTTGTGGCTTTTG	NM_001134.3	1583-1739
IL1-β	Interleukin-1beta	GCAGAAGTACCTGAGCTCGC	TCCATGGCCACAACAACTGA	NM_000576.3	91-302
TNF-α	Tumor necrosis factor alpha	GCAACAAGACCACCACTTCG	GATCAAAGCTGTAGGCCCCA	NM_000594.4	1020-1143
CXCL10	C-X-C Motif Chemokine Ligand 10	GCTTCCAAGGATGGACCACA	GCAGGGTCAGAACATCCACT	NM_001565.4	392-644
MRC1	Mannose receptor C type 1	CGTTCCTTTGGACGGATGGA	CCTCGTTTACTGTCGCAGGT	NM_002438.4	3174-3345
ABCC2	ATP binding cassette subfamily C member 2	TCAGACGACCATCCAAAACGA	GGGTCCAGGGATTTGTAGCAG	NM_000392.5	4630-4786
**ABCC4**	ATP binding cassette subfamily C member 4	ATTTATCGGAAGGCACTTCGT	CAAGGGCAGGAGAATGATTAGA	NM_001105515.3	657-887
**CYP1A2**	Cytochrome P450 family 1 subfamily A member 2	CCTTCGCTACCTGCCTAACC	CTCTAGGCCCCTTCTTGCTG	NM_000761.5	785-951
**CYP3A5**	Cytochrome P450 family 3 subfamily A member 5	CTCCTCTATCTATATGGGACCCG	TGACCTTCATACGTTCCCCAC	NM_000777.5	161-333
**SULT1A1**	Sulfotransferase family 1A member 1	CTGGAGAAGTTCATGGTCGGA	CCACGAAGTCCACGGTCTC	NM_001055.4	593-770
**SULT1B1**	Sulfotransferase family cytosolic 1B member 1	GCCTATGGTTCCTGGTTTACTC	AGTAATTCTTCCAGTCACCAGC	NM_014465.3	803-1082
**UGT1A1**	UDP-glucuronosyltransferase family 1 member A1	TGCGACGTGGTTTATTCCCC	AGGCTTCAAATTCCTGGGATAGTG	NM_012683.2	682-869
**UGT2B7**	UDP-glucuronosyltransferase family 2 member b7	AGGAGCTAAACACCTTCGGG	TGCTGGAATAAACTGAAGTAGTCTC	NM_001074.4	1460-1699
**GSTT1**	Glutathione S-transferase theta 1	ACGGGGACTTCACCTTGAC	GTACTCATCCACACGGGCAC	NM_000853.4	216-334

**GSTT2**	Glutathione S-transferase theta 2	CGACGCTCAAGGATGGTGA	CTCCTCCAGGGCCATGAGAT	NM_000854.5	228-580
**GSTA1**	Glutathione S-transferase alpha 1	CCTGAGGAAAAAGATGCCAA	GACTGGAGTCAAGCTCCTCG	NM_145740.5	402-582
**GSTA2**	Glutathione S-transferase alpha 2	TCCTTCTTCTGCCCTTTAGTC	GCTGGAAATAAGGCTAGAGTCAA	NM_000846.5	379-593
**GSTA4**	Glutathione S-transferase alpha 4	GTTGGTACAGACCCGAAGCA	GCTTTGTCCGTGACCCCTTA	NM_001512.4	246-492
**GSR**	Glutathione-disulfide reductase	GGCCGAAAACTTGCCCATC	ATCGGGGTAAAGCTCGTTGA	NM_000637.5	1179-1360
**GPX1**	Glutathione Peroxidase 1	GTCGGTGTATGCCTTCTCGG	TCTTGGCGTTCTCCTGATGC	NM_000581.4	117-340
**GPX2**	Glutathione peroxidase 2	TGAGAATGTGGCTTCGCTCTG	ACCCCCAGGACGGACATAC	NM_002083.4	118-298
**GPX4**	Glutathione peroxidase 4	CGCTGTGGAAGTGGATGAAG	AGAAATAGTGGGGCAGGTCC	NM_002085.5	475-642
**CASP3**	Caspase 3	TGCATACTCCACAGCACCTG	TCTGTTGCCACCTTTCGGTT	NM_004346.4	820-972
**CASP7**	Caspase 7	AGCCTGGGTTTTGACGTGAT	CCCCTAAAGTGGGCTGTCAAA	NM_001227.5	374-570
**DDIT**	DNA damage inducible transcript 3	ATGAACGGCTCAAGCAGGAA	GGGAAAGGTGGGTAGTGTGG	NM_001195053.1	750-897
**ATF4**	Activating Transcription Factor 4	GAAGCGATTTAACGAGCGCC	ATCTTGGTTCCTGCCACGTT	NM_001675.4	293-489
**ATF6**	Activating Transcription Factor 6	GTATCAGGAACTCAGGGAGTG	GCAGGTGATCCCTTCGAAAT	NM_001410890.1	1639-1767
**CAT**	Catalase	TCAGGTGCGGGCATTCTATG	TGATGAGCGGGTTACACGGA	NM_001752.4	1403-1724
**SOD1**	Superoxide dismutase 1	ACAAAGATGGTGTGGCCGAT	CGACTTCCAGCGTTTCCTGT	NM_000454.5	349-508
**SOD2**	Superoxide Dismutase 2	CTCAGGTTGGGGTTGGCTTG	TGCTCCCACACATCAATCCC	NM_000636.4	506-634
**NFE2L2**	NFE2 like bZIP transcription factor 2	GCGACGGAAAGAGTATGAGC	TACAAACGGGAATGTCTGCG	NM_006164.5	274-541
**GCLC**	Glutamate-Cysteine Ligase Catalytic Subunit	TCAATGGGAAGGAAGGTGTGT	TGGTTTGCGATAAACTCCCTCA	NM_001498.4	2027-2227
**GCLM**	Glutamate-Cysteine Ligase Modifier Subunit	AATCTTGCCTCCTGCTGTGTG	ACTCGTGCGCTTGAATGTCAG	NM_002061.4	832-991
**TXNRD1**	Thioredoxin Reductase 1	GGTGCTTGTGGCCTTTCTGA	GGACCCAGTACGTGAAAGCC	NM_182729.3	1365-1552
**HMOX1**	Heme oxygenase 1	GACTGCGTTCCTGCTCAACAT	GGCTCTGGTCCTTGGTGTCAT	NM_002133.3	690-766
**NQO1**	NAD(P)H Quinone Dehydrogenase 1	TCCAGAAAGGACATCACAGGT	ATGAACACTCGCTCAAACCA	NM_000903.3	275-486

Table S3. IC50 values (μM) for test compounds in different in vitro models.

	**HepG2**	**Co-culture 1.10**	**Co-culture 1.5**	**Co-culture 1.2**
TVX	675	531	529	74
LVX	>800	>800	>800	>800
TGZ	223	208	203	188
RGZ	>250	>250	>250	>250
